# Oral Mosapride Attenuates Gastrointestinal Dysmotility During Experimental Endotoxemia in Horses: A Randomized, Blinded Crossover Study

**DOI:** 10.3390/ani16193032

**Published:** 2026-09-26

**Authors:** Diego Duarte Varela, Ana Carolina Ribeiro Rosa, Lara Nunes Sousa, Eduarda Zancanaro Luvison, Ana Moutinho Vilella Machado, Marcella Procópio Valle Couto, Dietrich Pizzigatti, Luiz Eduardo Duarte de Oliveira, Fabíola de Oliveira Paes Leme, Livia Camargo Garbin, Heloísa de Paula Pedroza, Rafael Resende Faleiros, Armando Mattos Carvalho

**Affiliations:** 1 EQUINOVA Research Group, Department of Veterinary Medicine and Surgery, Veterinary School, Federal University of Minas Gerais (UFMG), Belo Horizonte 31270-901, Brazil; diegoduartevarela@gmail.com (D.D.V.); anariberosa@gmail.com (A.C.R.R.); eduardazluvison@gmail.com (E.Z.L.); ana.ufmg.vet@gmail.com (A.M.V.M.); livia.garbin@gmail.com (L.C.G.); faleirosufmg@gmail.com (R.R.F.); 2 Veterinary School, Federal University of Minas Gerais (UFMG), Belo Horizonte 31270-901, Brazil; marcellacouto1998@gmail.com (M.P.V.C.); oliveiraled@ufmg.br (L.E.D.d.O.); fabiola.ufmg@gmail.com (F.d.O.P.L.); 3 Equine Development Pole, Northeast Regional University Center (CENUR-Noreste) of the University of the Republic (UdelaR), Melo 37000, Uruguay; dietrich.pizzigatti.dvm@gmail.com; 4 Veterinary Clinical Sciences, School of Veterinary Medicine, Louisiana State University (LSU), Baton Rouge, LA 70803, USA; pheloisa@lsu.edu

**Keywords:** equine, lipopolysaccharide, hypomotility, 5-HT4 receptor, prokinetic, postoperative ileus

## Abstract

Endotoxemia is a major contributor to gastrointestinal hypomotility and postoperative ileus in horses. This study investigated whether oral mosapride retained prokinetic activity during acute lipopolysaccharide (LPS)-induced endotoxemia. Seven healthy adult horses were enrolled in a randomized, blinded, three-period crossover trial and received LPS alone, mosapride alone (MOSA), or LPS plus mosapride (LPS + MOSA), with a 14-day washout interval between periods. Mosapride was administered orally at 2 mg/kg, whereas endotoxemia was induced by an intravenous infusion of *Escherichia coli* O55:B5 LPS (0.03 µg/kg) over 30 min. Clinical variables, hematologic parameters, abdominal auscultation, and transabdominal ultrasonography were assessed from baseline to 6 h. LPS induced tachycardia, hyperthermia, leukocyte alterations, marked gastrointestinal hypomotility, gastric distension, and increased intestinal wall thickness. Mosapride alone increased gastrointestinal motility without clinically relevant abnormalities. During endotoxemia, mosapride partially restored gastrointestinal motility, reduced intestinal wall thickening and gastric distension, and was associated with a less pronounced hematologic inflammatory response than LPS alone. These findings indicate that oral mosapride retains clinically relevant prokinetic activity during acute experimental endotoxemia and support further investigation of its use as an adjunctive treatment for inflammation-associated gastrointestinal hypomotility in horses.

## Simple Summary

Endotoxemia is an important cause of gastrointestinal hypomotility in horses and contributes to gastric retention, bowel distension, and postoperative ileus. A clinically useful prokinetic should therefore remain effective during inflammatory gastrointestinal dysfunction, rather than only in healthy animals. In this randomized, blinded crossover study, seven healthy horses were evaluated under three experimental conditions: lipopolysaccharide (LPS) alone, mosapride alone, and LPS plus mosapride. Oral mosapride at 2 mg/kg increased intestinal motility in healthy horses and partially restored motility during endotoxemia. Compared with untreated endotoxemic horses, those receiving mosapride during endotoxemia had greater auscultatory and ultrasonographic motility, less intestinal wall thickening, lower gastric distension, and a less pronounced hematologic inflammatory response. These findings support further evaluation of oral mosapride as an adjunctive prokinetic in horses with inflammation-associated gastrointestinal hypomotility and those at risk of postoperative ileus.

## 1. Introduction

Endotoxemia and postoperative ileus (POI) remain major therapeutic challenges in equine internal medicine and surgery because both are associated with systemic inflammation and marked impairment of coordinated gastrointestinal propulsion. In horses with acute abdominal disease, reduced intestinal transit promotes luminal stasis, delayed gastric emptying, progressive distension, bowel wall stress, impaired absorption, and persistent inflammation, all of which may worsen prognosis and prolong recovery [1,2,3]. Endotoxemia is particularly relevant in this context because lipopolysaccharide (LPS) exposure disrupts gastrointestinal motility while simultaneously inducing cardiovascular, hematologic, thermoregulatory and inflammatory abnormalities [1,3].

Experimental studies have directly demonstrated the sensitivity of equine gastrointestinal motility to relatively low doses of endotoxin. Clark and Moore showed that slow intravenous infusion of a cumulative LPS dose of 0.03 µg/kg—the same cumulative dose used in the present study, although administered over 60 rather than 30 min—reduced cecal and right ventral colon contractile activity and decreased cecal blood flow in healthy horses [4]. King and Gerring subsequently demonstrated marked disruption of gastric, small-intestinal, and colonic motor patterns after low-dose LPS administration and showed that several of these changes could be reproduced by prostaglandin E_2_ and, to a lesser extent, prostacyclin, supporting a role for inflammatory mediators in endotoxin-associated dysmotility [5].

Available prokinetic options in horses remain limited. Traditional agents such as metoclopramide, cisapride, and lidocaine have been used in equine gastrointestinal dysmotility, but concerns remain regarding efficacy, availability, and adverse effects [2,6,7]. More recently, a controlled endotoxemia study showed that lidocaine failed to mitigate LPS-induced pain and hypomotility in horses, reinforcing the need for alternative compounds with a more specific pharmacologic rationale and more consistent experimental efficacy under inflammatory conditions [8].

Mosapride is a selective 5-hydroxytryptamine-4 (5-HT4) receptor agonist that enhances acetylcholine release from enteric cholinergic neurons and promotes coordinated gastrointestinal propulsion [6,9,10]. The relevance of this pharmacologic target in horses is supported by equine-specific evidence. Giancola et al. demonstrated extensive 5-HT4 receptor expression in equine myenteric and submucosal neurons and in extrinsic sensory fibers [11], whereas Prause et al. demonstrated 5-HT4 receptor expression and functional responses in tissues from the equine duodenum, ileum, and pelvic flexure [12]. Together, these studies provide anatomical and functional support for 5-HT4-mediated modulation of equine gastrointestinal motility.

Experimental evidence in horses has shown that mosapride increases small-intestinal electrical activity [13] and promotes both small-intestinal and cecal activity in a dose-dependent manner, with maximal responses generally reported at 1.5–2 mg/kg [14]. Mosapride has also been shown to facilitate gastric emptying in healthy horses [15] and has compared favorably with other prokinetic agents in studies assessing gastric, jejunal, and cecal motor activity [16]. Of particular clinical relevance, Okamura et al. demonstrated that oral mosapride at 1.5 mg/kg accelerated recovery of small-intestinal motor activity in horses following celiotomy and jejunocaecostomy [17]. Thus, the available equine literature extends beyond normal gastrointestinal physiology and provides preliminary evidence that mosapride remains active in the surgically manipulated intestine. Pharmacokinetic studies have also demonstrated rapid oral absorption and approximately dose-proportional exposure between 0.5 and 1.5 mg/kg in horses [18]. Dose-dependent prokinetic effects have been reported in donkeys [19], and mosapride has enhanced ruminal motility in cattle [20].

However, demonstrating prokinetic activity in healthy or surgically manipulated animals is insufficient to establish efficacy during inflammation-associated gastrointestinal dysfunction. Endotoxemia and postoperative ileus are multifactorial processes involving inflammatory mediator release, altered autonomic and enteric neural signaling, changes in intestinal perfusion, leukocyte recruitment, and impaired neuromuscular function [1,21,22]. Morphologic studies in experimentally endotoxemic horses have demonstrated ischemic changes in myenteric neurons together with edema, degeneration, and necrosis of intestinal smooth muscle, indicating that endotoxin-associated gastrointestinal dysfunction can involve both structural and functional alterations [23].

Increasing evidence also indicates that 5-HT4 receptor activation may influence the neuroimmune pathways involved in intestinal inflammation. Stakenborg et al. demonstrated that preoperative stimulation of enteric 5-HT4 pathways with prucalopride reduced intestinal inflammation and shortened POI through cholinergic mechanisms [24]. More directly relevant to mosapride, Tsuchida et al. showed that the drug attenuated postoperative motility impairment, leukocyte infiltration, and expression of IL-1β, IL-6, TNF-α, MCP-1, and inducible nitric oxide synthase in a rodent model of POI through a cholinergic pathway involving α7 nicotinic acetylcholine receptors [25]. Kimura et al. subsequently confirmed that the anti-inflammatory action of mosapride in experimental POI depended on 5-HT4 receptor activation and demonstrated differential cholinergic regulation of macrophage and neutrophil recruitment [26]. Although these findings cannot be directly extrapolated to horses, they provide a biologically plausible mechanism by which 5-HT4 agonism has effects beyond motor activity.

In another experimental study, mosapride increased intestinal transit, altered intestinal microbiota, and reduced bacterial translocation and circulating endotoxin in cirrhotic rats [27]. Notably, mosapride did not improve intestinal histologic damage or restore expression of the tight-junction proteins occludin and ZO-1 in that model [27]. These findings therefore support an association between improved motility and reduced systemic endotoxin burden rather than a direct barrier-restorative effect. Studies of experimental endotoxemia in horses have also demonstrated rapid and dynamic inflammatory responses, including characteristic leukocyte redistribution [28,29] and early upregulation of cytokines such as IL-1β, IL-6, IL-8, and TNF-α [30].

Therefore, the objective of the present study was to determine whether oral mosapride at 2 mg/kg could improve gastrointestinal motility in horses subjected to experimental LPS-induced endotoxemia and to evaluate clinically relevant secondary outcomes, including gastric distension, intestinal wall thickness, and hematologic indicators of systemic inflammation. We hypothesized that mosapride would not prevent the systemic endotoxemic response but would preserve or restore gastrointestinal motor activity and attenuate some manifestations of endotoxemia-associated gastrointestinal dysfunction.

## 2. Materials and Methods

### 2.1. Animals and Experimental Design

The study was conducted at the EQUINOVA Teaching and Development Center, located at the Experimental Farm of the Federal University of Minas Gerais (UFMG), Pedro Leopoldo, Minas Gerais, Brazil. The study protocol was approved by the UFMG Ethics Committee on the Use of Animals (protocol no. 274/2023; approved on 20 December 2023). Animal management and experimental procedures were conducted in accordance with national guidelines for the use of animals in research.

A randomized, blinded crossover design was used. Treatment order was determined by drawing lots before the experimental periods. All seven horses received each of the three treatments, with a minimum 14-day washout interval between periods. Only the study coordinator had access to treatment allocation. Investigators responsible for clinical evaluations and data recording were unaware of the treatment administered. Ultrasonographic recordings were subsequently analyzed by an experienced examiner who was blinded to both treatment allocation and horse identity.

Sample size was estimated using the online tool “Statistical Considerations for a Cross-Over Study Where the Outcome Is a Measurement” developed by Schoenfeld. The calculation was based on a pairwise treatment comparison within a crossover design. Estimates were derived from a previous crossover study in seven horses evaluating the effects of Buscopan^®^ compositum on gastrointestinal motility [31]. Based on descending duodenal contraction data from that study, the calculation assumed a two-sided significance level (α) of 0.05, a within-subject standard deviation of 1.2 contractions/3 min, a target power of 90%, and a minimum detectable treatment difference of 2.0 contractions/3 min. Under these assumptions, seven horses provided approximately 91% power to detect the specified treatment difference.

Seven healthy adult mixed-breed horses (four males and three females) were included. Mean age was 5.1 ± 3.4 years, and mean body weight was 307.6 ± 51.2 kg. Horses were considered clinically healthy based on physical examination, complete blood count, and hepatic and renal biochemical profiles, and none had a history of abdominal discomfort during the previous six months.

The horses were maintained in paddocks with Coast-cross grass and underwent a 10-day acclimation period before the start of the study. They received hay at 1% of body weight, a commercial maintenance concentrate containing 12% crude protein at 0.5% of body weight, and a protein-energy supplement, with water available ad libitum. Horses were not fasted before or during the experimental procedures. Rescue analgesia was available if clinically indicated; however, no horse required analgesic intervention during any experimental period.

### 2.2. Treatments and Endotoxemia Induction

The experimental treatments were as follows: (1) LPS alone, consisting of oral saline plus intravenous LPS infusion; (2) MOSA, consisting of oral mosapride without LPS, received a saline infusion for 30 min to simulate the LPS infusion; and (3) LPS + MOSA, consisting of oral mosapride plus intravenous LPS infusion.

Before each experimental period, a 14-gauge peripheral venous catheter was placed in a jugular vein following clipping and aseptic skin preparation. Horses were then positioned in stocks for clinical monitoring, intravenous infusions, and nasogastric intubation.

Mosapride citrate (10 mg tablets; Gastrial^®^, Gramón Bagó, Uruguay) was administered orally at 2 mg/kg via a nasogastric tube after dilution in 1000 mL of 0.9% sodium chloride solution. During the placebo periods, 1000 mL of 0.9% sodium chloride solution was administered orally using the same procedure.

Endotoxemia was induced during the LPS and LPS + MOSA periods using *Escherichia coli* O55:B5 lipopolysaccharide (L2880; Sigma-Aldrich, St. Louis, MO, USA) at 0.03 µg/kg. LPS was diluted in 500 mL of 0.9% sodium chloride solution and administered as an intravenous infusion over 30 min.

### 2.3. Clinical and Hematologic Assessments

Evaluations were performed at baseline (T0) and at T0.5, T1, T1.5, T2, T3, T4, T5, and T6 h. At each time point, heart rate, respiratory rate, rectal temperature, capillary refill time, mucous membrane characteristics (score: 0 = pale; 1 = pink/normally colored; 2 = congested/hyperemic), urine output (score: 0 = absent; 1 = present), fecal characteristics (score: 0 = absent; 1 = normal/present; 2 = soft/pasty; 3 = diarrheic), and the presence of an endotoxemic halo (score: 0 = absent; 1 = present) were recorded using standardized clinical evaluation forms.

Blood samples were collected into K3-EDTA tubes at each time point, immediately refrigerated, and submitted to the Veterinary Clinical Pathology Laboratory of MULTILAB-UFMG for hematologic analysis (Icounter 3D, Diagno Ltd., Belo Horizonte, Brazil).

### 2.4. Gastrointestinal Motility and Ultrasonography

Gastrointestinal motility was assessed by abdominal auscultation and transabdominal ultrasonography.

Abdominal auscultation was performed using a 3M™ Littmann^®^ CORE digital stethoscope (3M, St. Paul, MN, USA) in diaphragm mode. The device amplifies sounds within the 20–2000 Hz range, with optimized sensitivity between 100 and 500 Hz. Before auscultation, the abdominal hair was clipped and the skin was moistened with alcohol to reduce friction-related acoustic artifacts. Auscultation was performed sequentially in the left dorsal quadrant (LDQ), left ventral quadrant (LVQ), right dorsal quadrant (RDQ), and right ventral quadrant (RVQ), with recordings obtained for 2 min in each quadrant. Borborygmi were graded using a previously described semiquantitative scoring system [31]. Recordings containing substantial acoustic artifacts were repeated.

Transabdominal ultrasonography was performed using a SonoScape E2V veterinary ultrasound system equipped with a 5 MHz macroconvex transducer (SonoScape Medical Corp., Shenzhen, China). After identification of the appropriate anatomical region, the skin was cleaned with alcohol, and acoustic coupling gel was applied. Segmental gastrointestinal motility and wall thickness were evaluated using adapted ultrasonographic protocols based on previously described methods for motility assessment [8] and intestinal wall thickness measurement [32].

The transducer was positioned longitudinally for evaluation of sacculated large-intestinal segments and transversely for examination of the descending duodenum. Gastrointestinal contractions were recorded for 3 min in the cecum, right ventral colon (RVC), small colon, left ventral colon (LVC), right dorsal colon (RDC), and duodenum. Duodenal activity was identified by circular distension and contraction, cecal contractions by displacement of the cecal wall by >2 cm, and colonic contractions by changes in sacculation.

Gastrointestinal wall thickness was measured in the cecum, RVC, small colon, LVC, RDC, duodenum, and stomach. Gastric distension was estimated from the position of the caudal gastric margin and the number of intercostal spaces over which the stomach extended.

All ultrasonographic examinations were digitally recorded and stored for subsequent analysis. Three-minute recordings from each gastrointestinal segment were reviewed by an examiner experienced in equine gastrointestinal ultrasonography who was blinded to treatment allocation and horse identity.

### 2.5. Statistical Analysis

Statistical analyses were performed using GraphPad Prism version 10.4.1. Data were tested for normality using the D’Agostino–Pearson and/or Kolmogorov–Smirnov tests. Variables were analyzed using two-way analysis of variance (ANOVA) to assess the effects of time, treatment, and the time × treatment interaction, followed by Tukey’s test for multiple comparisons between means. For nonparametric data, the Friedman test was used to compare different time points within each treatment, and the Kruskal–Wallis test was used to compare treatments at the same time point, followed by Dunn’s test for multiple comparisons. For all analyses, statistical significance was set at *p* < 0.05 (5%).

## 3. Results

LPS administration elicited a clear systemic clinical response in both LPS-containing treatments, with changes generally more pronounced in the LPS group. Clinical variables remained comparatively stable during MOSA treatment, whereas LPS + MOSA generally showed an intermediate response (Figure 1).

Changes in mucous membrane color were more apparent in the oral mucosa than in the ocular mucosa. Ocular mucous membrane color changes were modest and occurred mainly in the LPS group during the early phase. Oral mucous membrane color changes were more pronounced, with the LPS group differing significantly from MOSA and, at selected time points, from LPS + MOSA.

Urine output, fecal characteristics, and endotoxemic halo also varied over time. The endotoxemic halo was observed most consistently in the LPS group, whereas LPS + MOSA showed a less pronounced and delayed pattern. These variables remained comparatively stable during MOSA treatment (Figure 2).

### 3.1. Hematologic Variables

#### 3.1.1. Leukogram

The leukogram showed marked changes after LPS administration. In the LPS group, total leukocyte counts decreased sharply during the early phase, with the nadir occurring between T1 h and T2 h, followed by gradual recovery. Neutrophils showed a similar biphasic pattern, with marked neutropenia early after LPS infusion followed by a progressive rebound later in the observation period.

LPS + MOSA showed the same overall trend, although leukopenia and neutropenia were generally less severe and the later rebound more moderate than in the LPS group. MOSA showed the most stable profile, with consistently higher leukocyte and neutrophil counts than the LPS-containing groups (Figure 3).

Lymphocyte counts decreased progressively in all groups but were most markedly reduced in LPS-containing treatments. Monocyte and eosinophil counts also changed over time, with LPS + MOSA often showing intermediate values between LPS and MOSA. Overall, the hematologic changes in LPS + MOSA were generally less pronounced than those in LPS alone.

#### 3.1.2. Erythrogram, Fibrinogen, and Platelets

Red blood cell count, hematocrit, and hemoglobin varied only modestly among treatments. Although some differences were observed, particularly in hemoglobin, these parameters showed less pronounced treatment-related variation than the leukogram. Fibrinogen showed more marked treatment-related changes. The LPS group showed an increase in fibrinogen concentration during the inflammatory phase, with significantly higher values than MOSA at multiple time points. Fibrinogen levels also increased in the LPS + MOSA group but remained generally lower than those observed in the LPS group. Platelet counts remained comparatively stable and showed less variation than leukocyte-related variables (Figure 4).

### 3.2. Gastrointestinal Assessment

#### 3.2.1. Auscultatory Gastrointestinal Motility

Abdominal auscultation revealed marked hypomotility in the LPS group across all four abdominal quadrants. In the RDQ, RVQ, LDQ, and LVQ, borborygmi decreased significantly from T0.5 h onward, reaching their lowest values generally between T1.5 h and T2 h. Although gradual recovery occurred thereafter, borborygmi scores remained below baseline for much of the observation period.

In the MOSA group, borborygmi scores increased above baseline during the early phase after mosapride administration and remained consistently higher than those in the LPS group. In LPS + MOSA, reductions in auscultatory motility were less pronounced and shorter in duration than in LPS. Recovery occurred earlier, and motility scores were consistently greater than those in untreated endotoxemic horses. At several time points, LPS + MOSA did not differ significantly from MOSA (Figure 5).

#### 3.2.2. Ultrasonographic Gastrointestinal Motility

Ultrasonographic showed segment-specific changes in gastrointestinal motility that were consistent with the auscultatory findings. In the LPS group, contraction counts decreased markedly after LPS infusion in all evaluated segments, including the cecum, right ventral colon, small colon, left ventral colon, right dorsal colon and duodenum. The greatest reduction occurred during the first 1–3 h after LPS administration.

MOSA showed the greatest contractile activity during the early period after treatment. LPS + MOSA showed intermediate but consistently higher values than LPS. In several segments, contraction counts in LPS + MOSA were significantly higher than those in LPS and approached those observed in MOSA during the later observation period (Figure 6).

#### 3.2.3. Gastrointestinal Wall Thickness and Gastric Distension

Intestinal wall thickness differed among treatments (Figure 7 and Figure 8). In the LPS group, wall thickness increased in several gastrointestinal segments, including the cecum, right ventral colon, left ventral colon, right dorsal colon, and duodenum. These changes were most evident during the early and intermediate phase after LPS administration.

Wall thickness remained comparatively stable during MOSA treatment. In LPS + MOSA, wall thickness was consistently lower than in LPS in multiple segments, particularly the right ventral colon, left ventral colon, right dorsal colon, and duodenum. At several time points, values in LPS + MOSA approached those in MOSA.

Gastric measurements also differed among treatments (Figure 8). Gastric distension and intercostal extension were greater in the LPS group than in MOSA, whereas LPS + MOSA showed lower values than LPS during the acute response to LPS.

## 4. Discussion

The present study showed that oral mosapride at 2 mg/kg retained a substantial prokinetic effect during acute experimental endotoxemia in horses. This was the main finding of the study. In addition to increasing gastrointestinal activity in healthy horses, mosapride administered concurrently with LPS was associated with higher auscultatory and ultrasonographic motility, less gastrointestinal wall thickening, and lower gastric distension than LPS alone. Several clinical and hematologic changes were also less pronounced with the combined treatment. Thus, the acute inflammatory challenge did not abolish the prokinetic effect of mosapride.

The response to LPS was consistent with previous experimental models of equine endotoxemia. Clark and Moore used the same cumulative LPS dose as the present study (0.03 µg/kg) and reported decreased cecal and right ventral colon contractility and reduced cecal blood flow, although the infusion was administered over 60 min rather than 30 min [4]. King and Gerring similarly demonstrated marked disruption of equine gastrointestinal motor patterns after LPS administration and implicated prostaglandin-mediated mechanisms in the acute motility response [5]. These findings are consistent with the marked hypomotility observed during the present LPS treatment.

The temporal clinical and hematologic responses also indicated successful induction of endotoxemia. Horses developed tachycardia, hyperthermia, acute leukopenia and neutropenia followed by recovery, together with marked gastrointestinal hypomotility. Lilliehöök et al. described a comparable early decrease in circulating neutrophils and monocytes during experimental equine endotoxemia, followed by later rebound [29]. At the molecular level, Nieto et al. demonstrated that IL-1α, IL-1β, IL-8, and TNF-α gene expression peaked approximately 60 min after LPS administration, whereas IL-6 peaked at approximately 90 min [30]. Thus, the most pronounced gastrointestinal alterations in the present experiment occurred during the expected acute inflammatory response to LPS.

The prokinetic response to mosapride was also consistent with previous equine studies. Sasaki et al. reported increased small-intestinal electrical activity after oral administration of 2 mg/kg [13], and subsequent dose–response work showed that 1.5–2 mg/kg promoted both small-intestinal and cecal activity [14]. Okamura et al. reported facilitated gastric emptying [15] and favorable effects on gastric, jejunal, and cecal motor variables compared with other prokinetic agents [16]. Of particular clinical relevance, mosapride at 1.5 mg/kg accelerated recovery of small-intestinal motor activity after celiotomy and jejunocaecostomy in horses [17]. The present study extends these observations by demonstrating a positive motor response during an acute endotoxemic challenge rather than only in healthy or postoperative, non-endotoxemic horses.

The segmental variation in response is also pharmacologically plausible. 5-HT4 receptors have been demonstrated in equine myenteric and submucosal neurons throughout several intestinal regions [11], and functional 5-HT4-mediated responses have been identified in the duodenum, ileum, and pelvic flexure [12]. Differences in receptor distribution, receptor reserve, local neural circuitry, and baseline motor patterns may therefore contribute to the magnitude and timing of responses among gastrointestinal segments. These equine data provide a more direct mechanistic basis for the present findings than extrapolation from human or laboratory-animal pharmacology alone.

However, mosapride did not prevent endotoxemia. Horses receiving LPS + MOSA continued to develop clinical and hematologic abnormalities, and gastrointestinal activity remained lower than in MOSA at several early time points. Mosapride should therefore be regarded as a prokinetic adjunct used in the presence of a systemic inflammatory insult rather than as an anti-endotoxin treatment. This distinction is important for clinical translation because treatment of the primary disease, endotoxin burden, inflammation, fluid deficits, and electrolyte disturbances would still be required in naturally occurring cases.

Lower intestinal wall thickness in LPS + MOSA than in LPS was an additional finding in several gastrointestinal regions. Experimental equine endotoxemia has previously been associated with edema of neural structures, ischemic changes in myenteric neurons, and edematous degeneration and necrosis of intestinal smooth muscle [23], supporting the possibility that endotoxin-associated dysfunction can involve structural changes in the intestinal wall. The increased wall thickness observed during the LPS period may therefore reflect acute mural changes accompanying the inflammatory and vascular response. However, the lower wall thickness in LPS + MOSA cannot be taken as direct evidence of an anti-inflammatory effect of mosapride because cytokines, tissue inflammatory infiltrates, vascular permeability, histology, and intestinal water content were not measured. In addition, a recent reliability study of ultrasonographic intestinal wall measurements in healthy horses found that 95% of repeated measurements fell within approximately 1 mm and concluded that differences smaller than 1 mm may result from measurement variability alone [32]. Accordingly, small between-treatment differences should be interpreted with caution, even when statistically significant. The ultrasonographic findings are therefore best described as treatment associated differences in intestinal wall thickness rather than evidence of reduced intestinal inflammation.

The gastric findings provide additional evidence of improved gastrointestinal function. Previous studies have shown that mosapride can facilitate gastric emptying in healthy horses [15,16]. In the present study, gastric distension was lower in LPS + MOSA than in LPS during part of the acute endotoxemic period. However, caudal gastric extension on ultrasonography is an indirect indicator of gastric distension and is not a direct measure of gastric emptying or luminal transit. Accordingly, these data indicate less gastric distension during endotoxemia but do not demonstrate enhanced gastric emptying.

The hematologic findings require similar caution. LPS + MOSA showed the same characteristic early response to LPS, but several later abnormalities were less pronounced than in LPS alone. This raises the possibility that effects associated with mosapride extended beyond contractile activity. Such an effect is biologically plausible: in rodent POI, mosapride reduced leukocyte infiltration and expression of several inflammatory mediators through 5-HT4-dependent cholinergic pathways [25,26], while prucalopride has shown analogous neuroimmune effects in experimental and translational studies [24]. However, the present experiment did not quantify cytokines or tissue leukocyte recruitment and therefore cannot establish that this mechanism occurred in horses. These findings indicate a less pronounced systemic hematologic response associated with treatment but do not establish immunomodulation.

The study by Xu et al. provides indirect support for a link between intestinal motility ans systemic endotoxin burden. In cirrhotic rats, mosapride increased intestinal transit while reducing bacterial translocation and circulating endotoxin [27]. However, it did not improve intestinal histologic injury or tight-junction protein expression [27], arguing against direct restoration of epithelial barrier integrity. If a similar relationship exists in horses, improved gastrointestinal motility could theoretically reduce luminal stasis and modify downstream gastrointestinal–systemic interactions, but this remains speculative and requires direct investigation.

Evidence for lidocaine in experimental equine endotoxemia is mixed. In a recent intravenous LPS model, lidocaine did not significantly improve auscultatory or ultrasonographic hypomotility and failed to mitigate LPS-induced abdominal pain [8]. By contrast, Peiró et al., using intraperitoneal LPS, reported that lidocaine reduced clinical scores and peritoneal TNF-α activity, although it did not prevent inflammatory cell infiltration [28]. Under the present experimental conditions mosapride produced a clear positive signal for gastrointestinal motor endpoints, but this was not a direct head-to-head comparison and does not establish superiority of mosapride over lidocaine in clinical POI.

These findings may also be relevant to postoperative ileus. POI is strongly associated with small-intestinal disease and other perioperative risk factors in horses [2], and its pathogenesis includes both neurogenic and inflammatory components [21,22]. Previous evidence that mosapride promoted recovery of intestinal activity after jejunocaecostomy [17], together with the present finding that its prokinetic activity persisted during endotoxemia, provides a rationale for further evaluation in horses undergoing colic surgery. The anti-inflammatory effects of mosapride demonstrated in rodent POI models [25,26] also provide a mechanistic hypothesis that could be specifically tested in equine postoperative patients.

Several limitations should be acknowledged. The experimental model consisted of a controlled, acute, and reversible LPS challenge in otherwise healthy horses. Naturally occurring colic and postoperative ileus involve additional factors, including ischemia–reperfusion injury, surgical manipulation, pain, anesthetic drugs, electrolyte and acid–base disturbances, hypovolemia, and variable endotoxin exposure. Efficacy in this model therefore cannot be assumed to translate directly to clinical POI. The sample size was modest, although the randomized crossover design reduced interindividual variability by allowing each horse to serve as its own control.

The 6 h observation period limited the assessment to acute endotoxemia, and increased intestinal contractile activity does not necessarily indicate effective propulsion of ingesta. Although auscultation and ultrasonography demonstrated enhanced gastrointestinal motility following mosapride administration, intestinal transit was not directly measured. Future studies combining ultrasonographic assessments with validated transit markers or scintigraphy could determine whether these prokinetic effects translate into effective gastrointestinal propulsion and functional restoration of intestinal transit.

The oral route is clinically practical but may be unsuitable in horses with severe gastric reflux or marked proximal gastrointestinal stasis, delayed gastric emptying may impair oral mosapride absorption and compromise its efficacy, while administration of additional fluid may be contraindicated. Therefore, our findings should not be extrapolated to horses with severe postoperative ileus or persistent gastric reflux. Available equine pharmacokinetic data extend only to oral doses of 1.5 mg/kg [18], whereas the present study used 2 mg/kg. Previous pharmacodynamic studies support 2 mg/kg as an effective dose [13,14,16], but systemic exposure at this dose has not been directly characterized in horses. Repeated-dose pharmacokinetic and safety studies would therefore be useful before broader clinical application.

Finally, because cytokines, intestinal histology, tissue leukocyte recruitment, permeability, the microbiome, and intestinal perfusion were not evaluated, it was not possible to determine whether the differences in wall thickness and systemic hematologic variables resulted directly from neuroimmune modulation or secondarily from improved gastrointestinal function. Future studies incorporating these endpoints would be valuable for testing the cholinergic anti-inflammatory mechanism suggested by experimental 5-HT4 receptor research [24,25,26].

Overall, oral mosapride at 2 mg/kg maintained substantial prokinetic activity during acute experimental endotoxemia and was associated with greater motor activity, less gastric distension, and lower intestinal wall thickness than LPS alone. The study does not establish mosapride as an anti-inflammatory or anti-endotoxin therapy; rather, it shows that 5-HT4 receptor-mediated prokinetic activity remains functional during an acute inflammatory challenge. Together with previous evidence of postoperative activity in horses and neuroimmune effects of 5-HT4 stimulation in other species, these findings support controlled clinical investigation of mosapride in naturally occurring equine gastrointestinal disease and POI.

## 5. Conclusions

Oral mosapride at 2 mg/kg retained prokinetic activity during acute experimental LPS-induced endotoxemia in horses. Compared with LPS alone, mosapride administration was associated with greater gastrointestinal motor activity, lower gastric distension, and lower intestinal wall thickness, although it did not prevent the systemic clinical and hematologic response to LPS.

These findings indicate that 5-HT4 receptor-mediated prokinetic activity remains functional during an acute inflammatory challenge and support further investigation of mosapride as an adjunctive prokinetic in horses with inflammation-associated gastrointestinal hypomotility. Controlled studies in naturally occurring gastrointestinal disease and postoperative ileus are needed to determine clinical efficacy, optimal dosing regimens, and the safety of repeated administration.

## Figures and Tables

**Figure 1:**
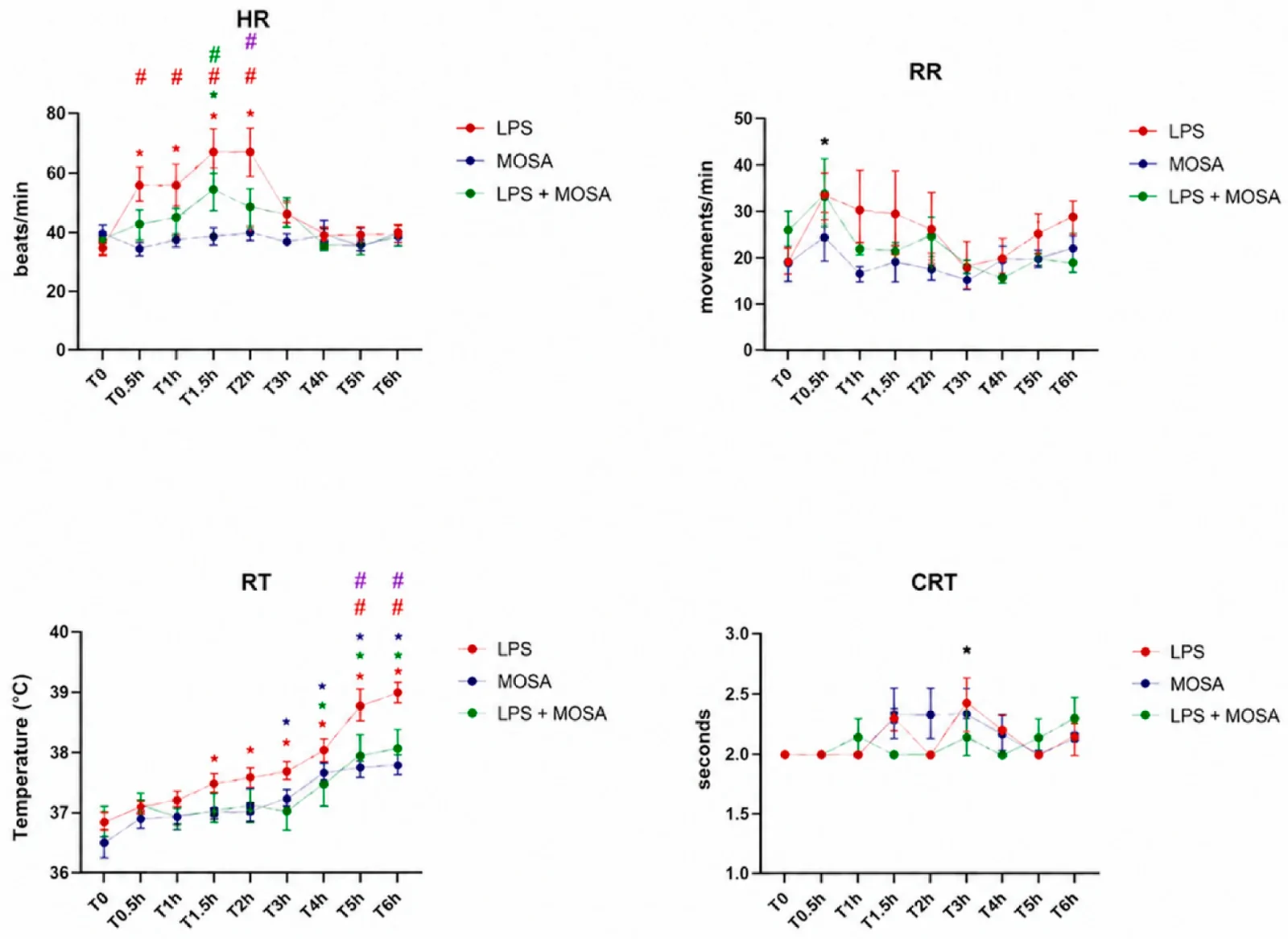
Heart rate (HR), respiratory rate (RR), rectal temperature (RT), and capillary refill time (CRT) in horses assigned to lipopolysaccharide (LPS), mosapride alone (MOSA), or LPS plus mosapride (LPS + MOSA) from T0 to T6 h. Data are presented as mean ± standard error. Colored hash symbols (#) indicate significant differences between treatments at the same time point (*p* < 0.05): purple #, LPS + MOSA vs. LPS; green #, LPS + MOSA vs. MOSA; red #, LPS vs. MOSA. Asterisks (*) indicate significant differences from T0 within the same treatment (*p* < 0.05).

**Figure 2:**
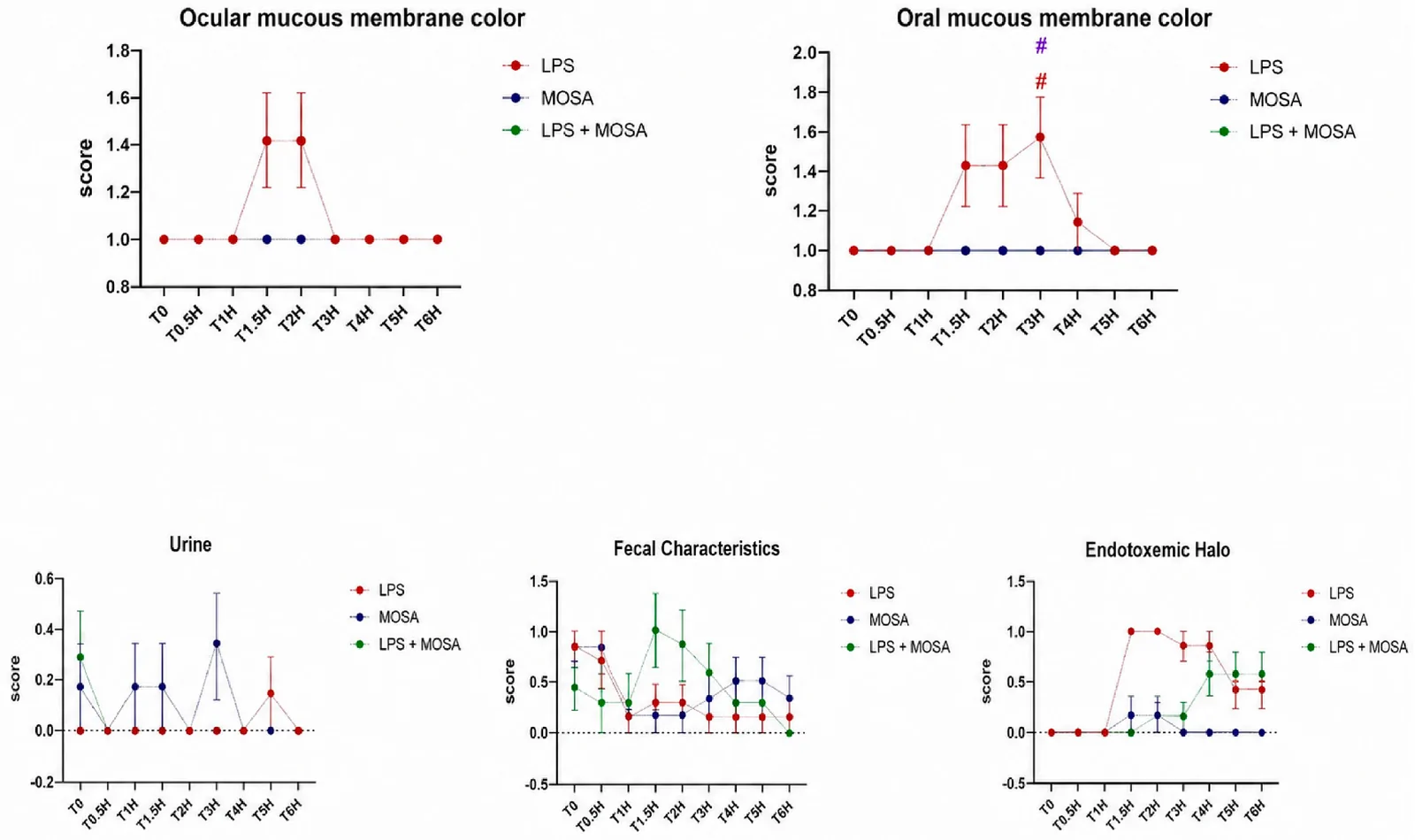
Ocular and oral mucous membrane color (score: 0 = pale; 1 = pink/normally colored; 2 = congested/hyperemic), urine output (score: 0 = absent; 1 = present), fecal characteristics (score: 0 = absent; 1 = normal/present; 2 = soft/pasty; 3 = diarrheic), and endotoxemic halo (score: 0 = absent; 1 = present) in horses assigned to lipopolysaccharide (LPS), mosapride alone (MOSA), or LPS plus mosapride (LPS + MOSA) from T0 to T6 h. Data are presented as mean ± standard error. Colored hash symbols (#) indicate significant differences between treatments at the same time point (*p* < 0.05): purple #, LPS + MOSA vs. LPS; red #, LPS vs. MOSA.

**Figure 3:**
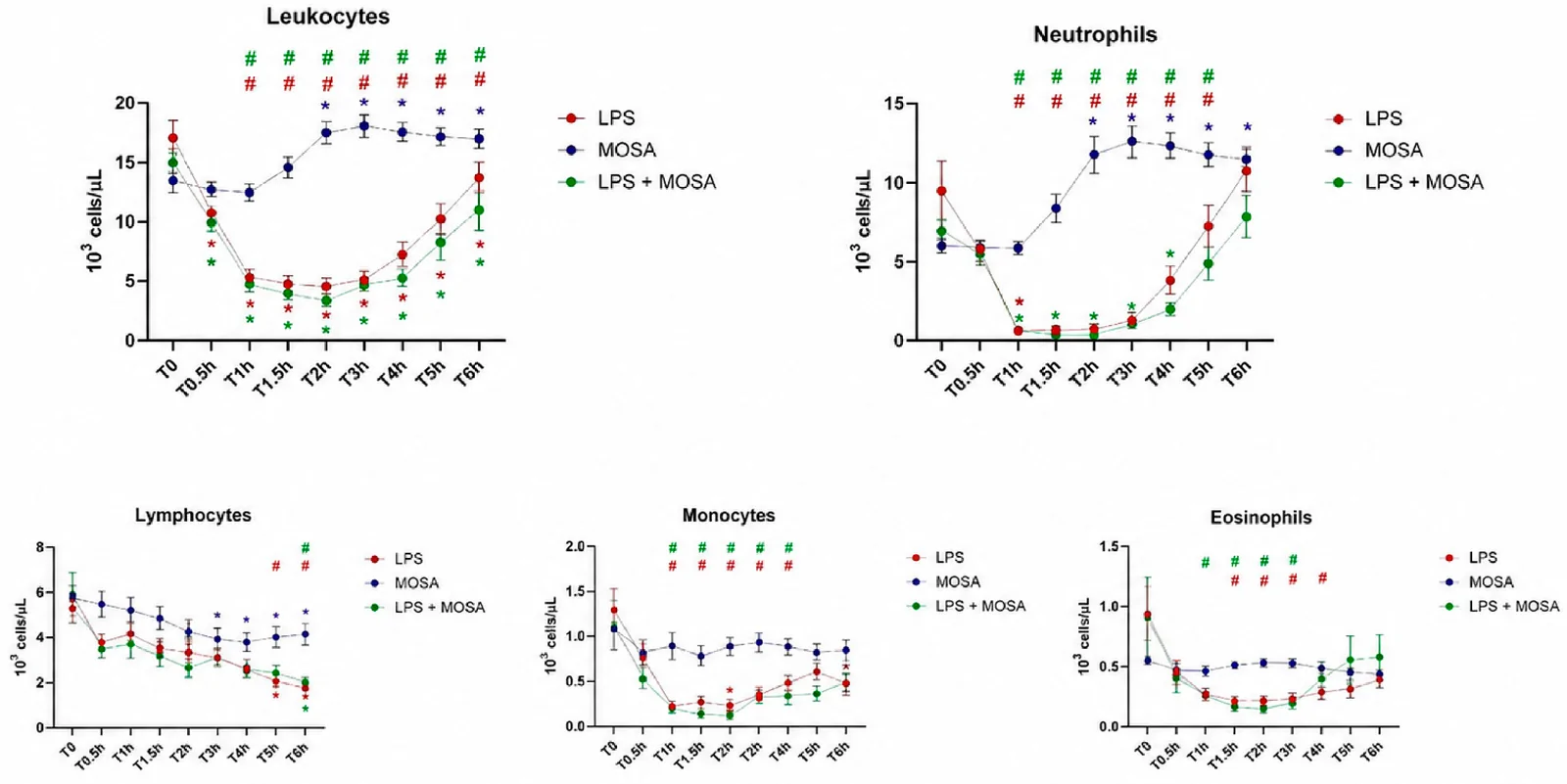
Total leukocytes, neutrophils, lymphocytes, monocytes, and eosinophil count in horses assigned to lipopolysaccharide (LPS), mosapride alone (MOSA), or LPS plus mosapride (LPS + MOSA) from T0 to T6 h. Data are presented as mean ± standard error. Colored hash symbols (#) indicate significant differences between treatments at the same time point (*p* < 0.05): green #, LPS + MOSA vs. MOSA; red #, LPS vs. MOSA. Asterisks (*) indicate significant differences from T0 within the same treatment (*p* < 0.05).

**Figure 4:**
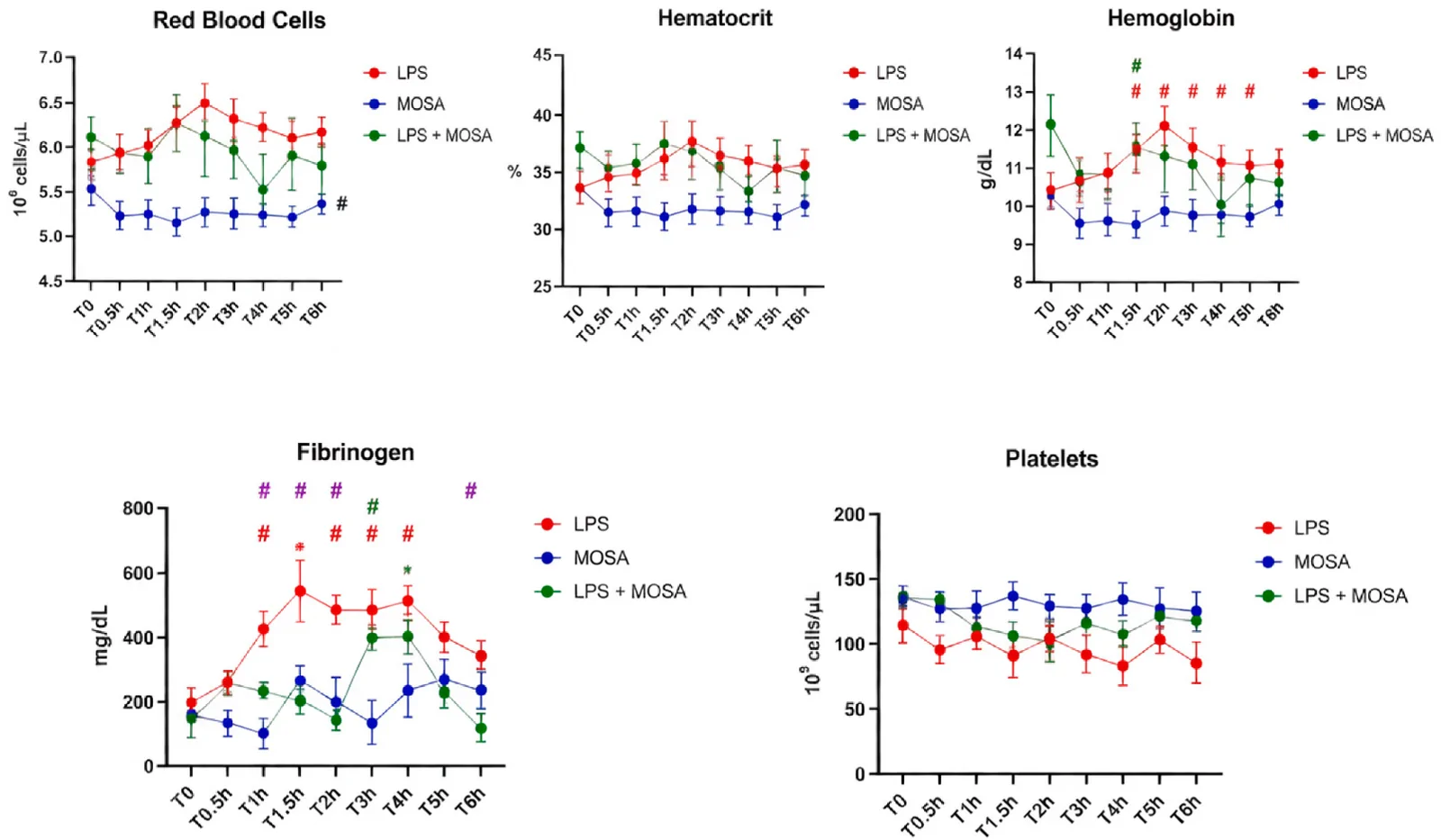
Red blood cell parameters, fibrinogen concentration, and platelet count in horses assigned to lipopolysaccharide (LPS), mosapride alone (MOSA), or LPS plus mosapride (LPS + MOSA) from T0 to T6 h. Data are presented as mean ± standard error. Hash symbols (#) indicate significant treatment differences (*p* < 0.05). Black # indicates an overall treatment effect independent of time; purple #, LPS + MOSA vs. LPS at the same time point; green #, LPS + MOSA vs. MOSA at the same time point; and red #, LPS vs. MOSA at the same time point. Asterisks (*) indicate significant differences from T0 within the same treatment (*p* < 0.05).

**Figure 5:**
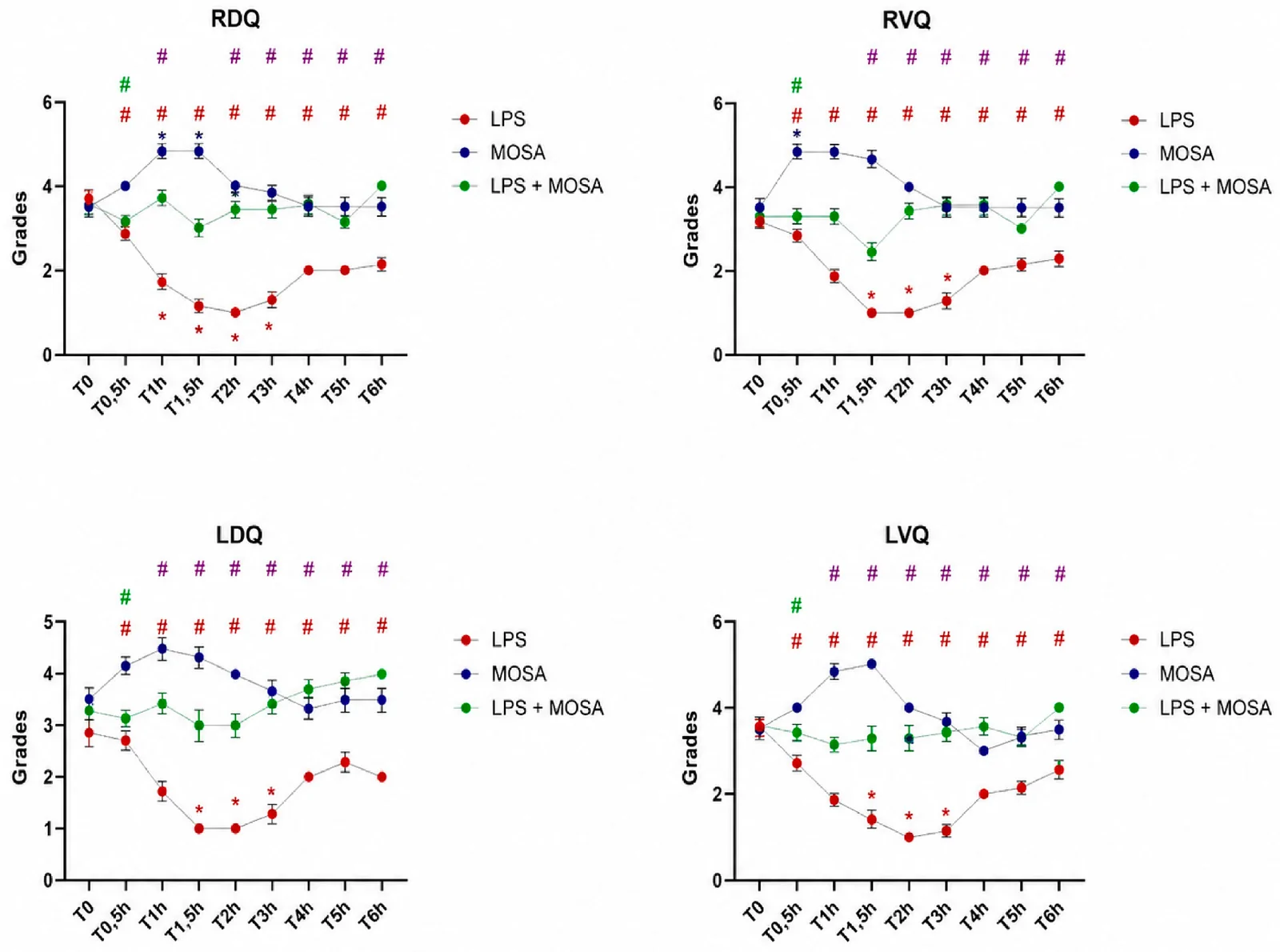
Borborygmi scores in the left dorsal (LDQ), right dorsal (RDQ), left ventral (LVQ), and right ventral (RVQ) abdominal quadrants in horses assigned to lipopolysaccharide (LPS), mosapride alone (MOSA), or LPS plus mosapride (LPS + MOSA) from T0 to T6 h. Data are presented as mean ± standard error. Colored hash symbols (#) indicate significant differences between treatments at the same time point (*p* < 0.05): purple #, LPS + MOSA vs. LPS; green #, LPS + MOSA vs. MOSA; red #, LPS vs. MOSA. Asterisks (*) indicate significant differences from T0 within the same treatment (*p* < 0.05).

**Figure 6:**
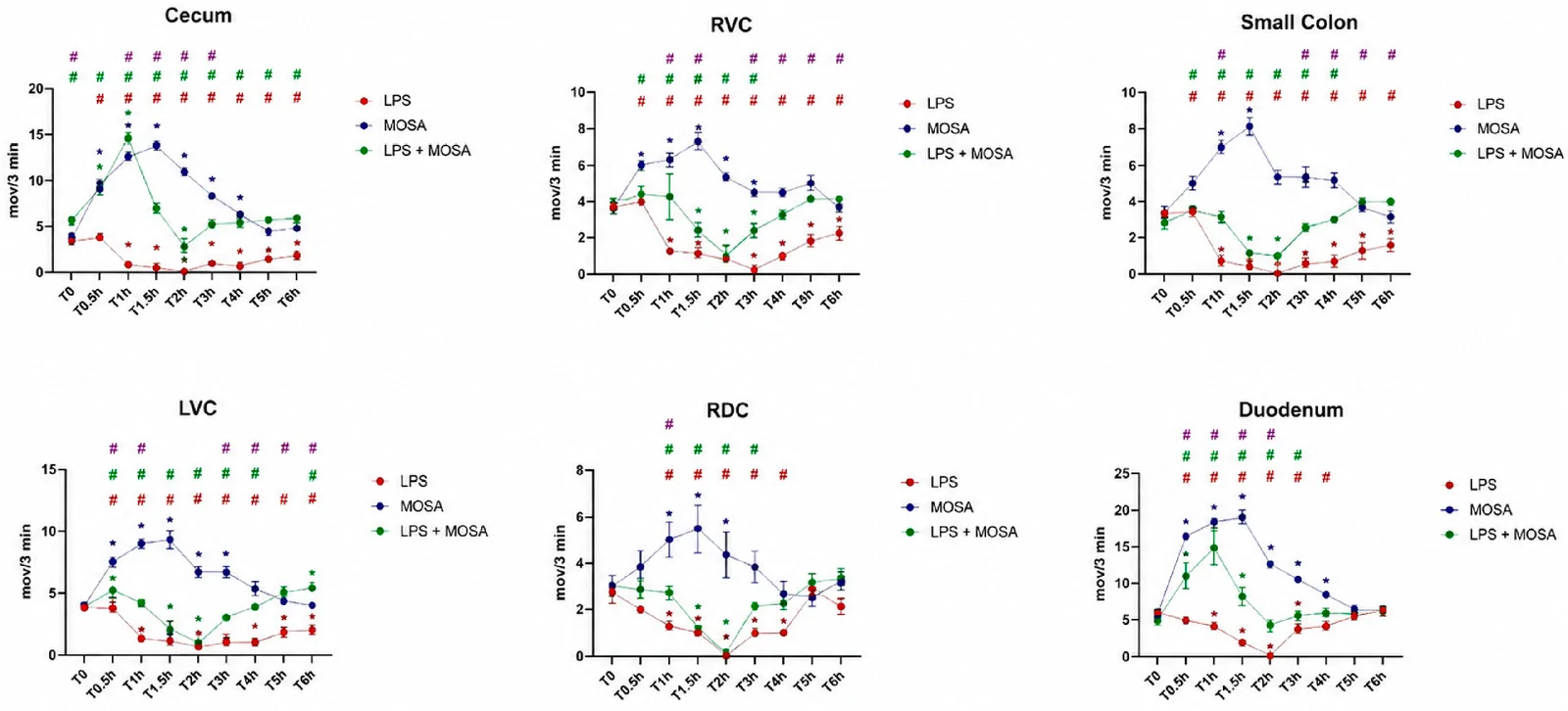
Ultrasonographic gastrointestinal motility during the experimental period. Contraction counts (movements/3 min) were recorded in the cecum, right ventral colon (RVC), small colon, left ventral colon (LVC), right dorsal colon (RDC), and duodenum in horses assigned to lipopolysaccharide (LPS), mosapride alone (MOSA), or LPS plus mosapride (LPS + MOSA) from T0 to T6 h. Data are presented as mean ± standard errors. Colored hash symbols (#) indicate significant differences between treatments at the same time point (*p* < 0.05): purple #, LPS + MOSA vs. LPS; green #, LPS + MOSA vs. MOSA; red #, LPS vs. MOSA. Asterisks (*) indicate significant differences from T0 within the same treatment (*p* < 0.05).

**Figure 7:**
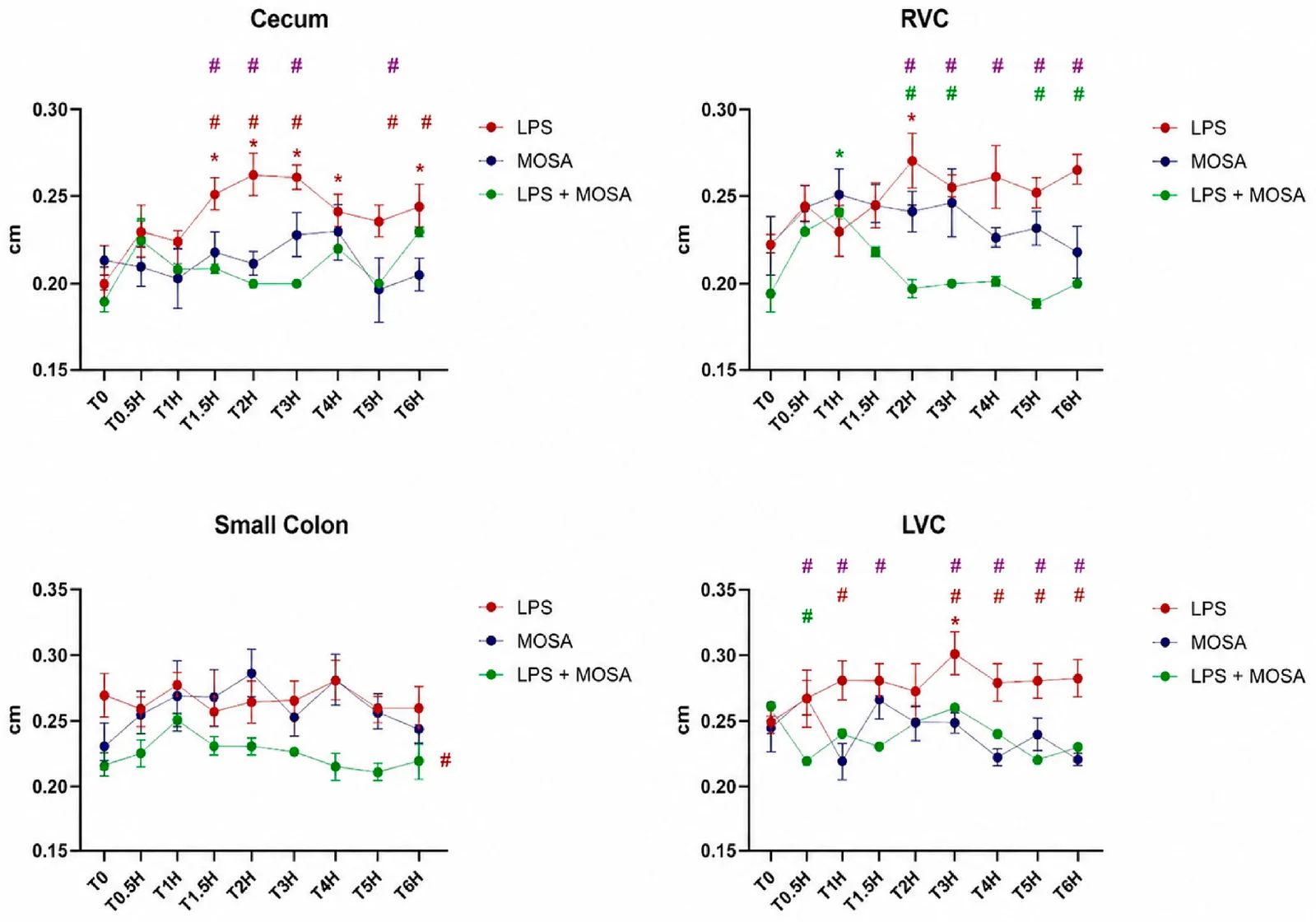
Intestinal wall thickness during the experimental period. Wall thickness (cm) was measured in the cecum, right ventral colon (RVC), small colon, and left ventral colon (LVC) in horses assigned to lipopolysaccharide (LPS), mosapride alone (MOSA), or LPS plus mosapride (LPS + MOSA) from T0 to T6 h. Data are presented as mean ± standard error. Colored hash symbols (#) indicate significant differences between treatments at the same time point (*p* < 0.05): purple #, LPS + MOSA vs. LPS; green #, LPS + MOSA vs. MOSA; red #, LPS vs. MOSA. Asterisks (*) indicate significant differences from T0 within the same treatment (*p* < 0.05).

**Figure 8:**
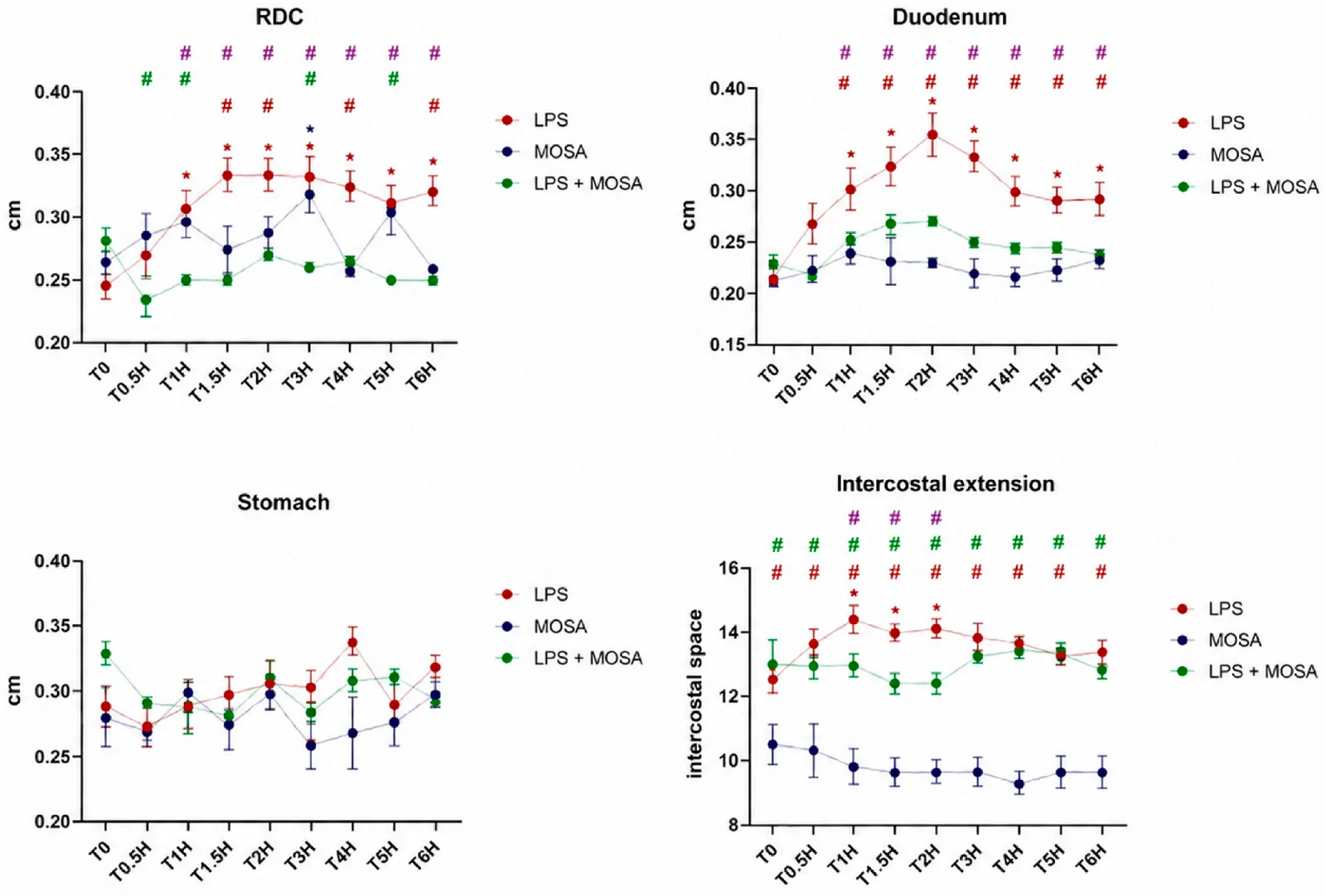
Segmental intestinal wall thickness and gastric measurements during the experimental period. Wall thickness was measured in the right dorsal colon (RDC), duodenum, and stomach, and gastric intercostal extension was assessed in horses assigned to lipopolysaccharide (LPS), mosapride alone (MOSA), or LPS plus mosapride (LPS + MOSA) from T0 to T6 h. Data are presented as mean ± standard errors. Colored hash symbols (#) indicate significant differences between treatments at the same time point (*p* < 0.05): purple #, LPS + MOSA vs. LPS; green #, LPS + MOSA vs. MOSA; red #, LPS vs. MOSA. Asterisks (*) indicate significant differences from T0 within the same treatment (*p* < 0.05).

## Data Availability

The raw data supporting the findings of this study are provided in the Supplementary Materials as Data S1. Further inquiries can be directed to the corresponding author.

## Supplementary Materials

The following supporting information can be downloaded at https://www.mdpi.com/article/10.3390/ani16193032/s1, Data S1. Individual raw data obtained from horses throughout the experimental study. The dataset includes gastrointestinal ultrasonographic evaluation, intestinal auscultation scores, physical examination parameters, complete blood count (CBC) variables, and gastrointestinal wall thickness measurements for the LPS, mosapride (MOSA), and mosapride plus LPS (MOSA + LPS) treatments at the different experimental time points.

## Abbreviations

LPS	Lipopolysaccharide
MOSA	Mosapride alone
LPS + MOSA	LPS plus mosapride
POI	Postoperative ileus
5-HT4	5-hydroxytryptamine-4
UFMG	Federal University of Minas Gerais
HR	Heart rate
RR	Respiratory rate
RT	Rectal Temperature
CRT	Capillary refill time
RDQ	Right dorsal quadrant
RVQ	Right ventral quadrant
LDQ	Left dorsal quadrant
LVQ	Left ventral quadrant
T	Time
RVC	Right Ventral Colon
LVC	Left Ventral Colon
RDC	Right Dorsal Colon
